# A Systematic Review of the Management of Incidental Findings in Genomic Research

**DOI:** 10.20541/beonline.2016.0006

**Published:** 2016-11-22

**Authors:** Cornelius Ewuoso

**Affiliations:** Department of Philosophy, Stellenbosch University, Stellenbosch, Western Cape, South Africa

**Keywords:** Incidental findings, Genome, Genomic research

## Abstract

Information empowerment has been the greatest gain of genomics, yet it also poses serious threat to its survival, especially when the information is incidental. There may be an emerging consensus that actionable incidental findings be returned. But this has not been supported by any systematic review. Future directions are equally missing. These are significant gaps.

To fill these gaps, an online search on PubMed and Genetics in Medicine website was conducted between 20th of August to 23rd of October, 2013; combining certain filters and phrases, such as ‘return incidental findings’. Nineteen (19) articles were selected from an avalanche of results, and reviewed. The review confirms a majority support for return of clinically actionable findings. The result also shows that the support represents views of Northern Americans. Critical contributions of Africans, Asians and Europeans are missing in this discourse. I recommended studies in this direction.

## Introduction

There is no denying the fact that genomics is redefining medicine in a whole new way. One key word describes the radical transformation which genomics is causing in medicine: Information-empowerment. This is directed at a two-fold end: predictive and preventive. Genomics is empowering physicians to find genetic alterations that make individuals susceptible to certain diseases such as cancer, diabetes or Down's syndrome. This novel endeavor has also offered humans the prospects of one day having treatment designed to help individuals overcome health anomalies. The extent of information that could be generated, using different technologies and methods, is unquantifiable.

Information-empowerment has been the greatest gain of genomics, yet it also poses the greatest threat to the survival of genomic research, especially when the information is not directly related to the aim of the study or is incidental. An incidental or ancillary finding is an information not directly intended and often beyond the scope of the study (Wolf et al., 2008b, Wolf et al., 2008a). Incidental finding largely generates controversy, in genomic research, when it is clinically significant; that is, when it is a finding about a research subject who is at a high risk of future preventable or manageable health problem. No doubt there are other reasons, such as misattributed paternity, why incidental finding may generate controversy. For example, owing to the sense of heritage, an incidental finding of misattributed paternity would be treated differently within Nigerian setting. Since such finding may quickly lead to loss of identity, loss of rights to inherit, stigmatization and expulsion from the community. The individual would be labeled a bastard. Such remarks have been known to cause great psychological distress for individuals. Sequel to these reasons, many Nigerians would generally not favor disclosure of incidental findings with misattributed paternity. And if they accidentally learn of this, such information would be guarded in secret, away from the ears of any third party.

But the frequencies of misattributed paternity in genomic research are relatively low when compared to clinically actionable findings (Booth et al., 2010). When clinically actionable findings arise, how should they be managed^1^? There claims by some theorists (Wolf et al., 2008b, Wolf, 2008, Wolf et al., 2008a, Wolf et al., 2012, Wolf, 2012) that a significant number of people want clinically significant findings returned to participants. But, this is not supported by any systematic review. Our examination of existing literatures shows that there is need to carefully reflect on the negative and positive implications of returning incidental findings to participants. These gaps are significant, since the current techniques and future technologies used in genomic research would continue to yield massive information, and some would be incidental (Wolf et al., 2008b). Present studies conjecture an incidental finding of misattributed paternity at a prevalence of 10% for general population; an incidental finding between 13% to 84% of brain magnetic imaging scans (Milstein, 2008) and an incidental finding of extracolonic lesions in between 15% to 89% of participants (Krier and Green, 2013, Morris et al., 2009, Wolf et al., 2008b).

This study would contribute to bridging these gaps, through a systematic review of current studies on incidental findings. In the final analysis, this study will make recommendations for future studies. First, we examine the challenges around managing incidental findings.

## Challenges around Incidental findings Management: Context and Debate

Research is commonly defined as a systematic investigation, including research development, testing and evaluation, designed to develop or contribute to generalizable knowledge(Levine, 2003). Research is equally either therapeutic if it provides direct benefit for the research participants, or non-therapeutic if it does not provide any direct healthcare benefits for the research participants. Generally, research aims at creating generalizable knowledge.

Research is to be distinguished from clinical care, which is oriented towards providing health benefits to the patients. Following this important distinction between research and clinical care, some scholars such as Solberg & Steinsbekk (2012) argue that researchers, unlike physicians, are not necessarily required to act for the health benefits of research participants. Unlike physicians who have a duty to follow up on a patient's health, there exists no relationship between researcher and research volunteers similar to doctor/patient relationship in the clinical context^2^. Some scholars (Bledsoe et al., 2012, Clayton and McGuire, 2012, Ossorio, 2012) also add that returning incidental findings to research participants would encourage therapeutic misconception; make the research enterprise financially burdensome; physically impracticable; harmful to research participants; and finally, may raise legal liabilities. Re-identifying research participants for the purpose of re-contacting them to offer them findings, Bledsoe and colleagues (2012) add, puts research participants at risks of losing their privacy, and confidentiality of data. These scholars also opine that unique issues may arise in retuning incidental findings to special population such as pregnant women following prenatal diagnosis, relatives of deceased participants and participants lacking capacity. Incidental findings, particularly those predictive of future illnesses may also create anxiety in participants and families, cause psychological harm to relatives and third parties. Breach of trust, respect for participants' privacy, the duty to warn versus right to not/know, may equally create legal issues in incidental findings management.

Other scholars (Lockhart et al., 2012, Wolf et al., 2012, Wolf et al., 2008a, Wolf et al., 2008b, Zawati and Knoppers, 2012), however, disagree that researchers do not have a general duty to return incidental findings. According to these scholars, research participants have a right to be informed about these findings given that certain conditions are fulfilled. They (Lockhart et al., 2012, Zawati and Knoppers, 2012) propose five criteria, for the general duty to return incidental findings:

- The findings are analytically valid
- Returning them to the donor comports with applicable law
- The donor has been offered that option of consenting to return of individual findings and has opted to receive them
- The findings reveal an established and substantial risk of (A) a serious health condition, or (B) a serious condition of reproductive importance
- The findings are clinically actionable

Returning clinically significant information, they conclude, would demonstrate the researcher's concern for research subject's welfare and autonomy, and strengthens the fiduciary relationship between the researcher and the research subject. Other scholars base the obligation to return incidental findings on other duties: duty of reciprocity and beneficence (Illes et al., 2006, Illes et al., 2008); respect for persons (Shalowitz and Miller, 2005); and the duty to rescue (Ulrich, 2013).

Following the above, the web of difficulties around how to manage incidental finding in genomic research, as Wolf and colleagues observed (Wolf, 2008)(Wolf, 2008), is “a ferocious tangle of science, medicine and ethics”. Scholars are certainly divided on how to manage incidental findings when they arise in research, following various reasons. Some scholars (Wolf et al., 2008a, Wolf, 2012) believe that there is now an emerging consensus in the academia that clinically significant incidental findings should be returned to participants. But this is not supported by any systematic review. In what follows, I shall attempt a systematic review of empirical studies on how to manage incidental findings.

## Method and Materials

A literature search was carried out between the 20^th^ of August to 23^rd^ of October, 2013, using PubMed search engine http://www.ncbi.nlm.nih.gov/pubmed/ and in Genetics in Medicine website http://www.nature.com/gim/journal/vaop/ncurrent/index.html (official journal of the American College of Medical Genetics and Genomics), to identify potentially relevant English publications. A combination of unique phrases - ‘incidental findings’, incidental findings genomic research’, ‘return incidental findings’, ‘return genomic results’, ‘managing incidental findings’, ‘reporting incidental findings’- suggested in published articles such as those published by Wolf and colleagues (2012, Wolf et al., 2008b, Wolf et al., 2008a, Wolf, 2012) were entered in both websites. The articles were not restricted to any particular years. Certain filters such as open and free, were also selected in Genetics in Medicine website. Article types were researches, and they were sorted by relevance to the unique phrases entered into the search engine. Only ‘empirical studies’, which examined how scientists and other stakeholders would handle incidental findings, or in any way explored participants' view on incidental findings management were selected from both websites. Other studies such as opinions and commentaries were excluded. This study was approved by the Ethical Committee of the University of Ilorin Teaching Hospital, Kwara State, Nigeria.

## Data Extraction Process

Using the filters identified and the criteria mentioned above, 8 articles were selected from official journal of the American College of Medical Genetics and Genomics and 13 articles were selected, using PubMed search engine. They were all carefully entered into EndNote database (version X6; Thomson Reuters). 1 duplicate reference (Green et al., 2012a) was removed. Two reviewers were asked to review the 20 studies. The studies were reviewed based on whether they were empirical studies or not. The two reviewers were unanimous in accepting 19 studies for review and dropping 1 (Wolf et al., 2008a), for not being an empirical study. All 19 articles, published in English language, were reviewed. Find the general characteristics of the articles below in the Table on General Characteristics:

The reviewed studies varied from studies involving human subjects (n=15) to studies involving documents and guidelines (n=4) for managing incidental findings. Majority (n=12) of the studies were carried out within non-clinical setting, that is, in a setting where there exists no physician/patient relationship between the researcher and the research participants.

To ensure standardization and aid pattern recognition, a data extraction form was developed. The form was piloted with three random studies. Changes were made to the form as a result of the pilot test^3^. All authors with known email addresses (18 authors) were re-contacted for a second review of the extracted data from their studies. Seven authors responded (response rate at 38.9%). Minor changes, relating to source of funding, population description etc., were made as a result of the second review. Using this data extraction form, information such as author(s), title, country of origin, study aims, participant description, year of publication, attitudes toward incidental findings, perspectives, field of genomic research, etc., were extracted and entered into STATA (version 12) for detailed description. This study reviewed all 19 studies. The studies were conducted largely in the United States of America (n=16) and Canada (n=3). The 19 studies examined views of IRB chairs, researchers, geneticists and genetic personnel, public and patients towards incidental findings

## Result

The result of the systematic review are hereby presented under the following headings:

- Understanding of incidental findings in Genomic Research
- Incidental findings management preference
- Conditions for returning incidental findings
- Factors affecting return of Incidental findings
- Who should return incidental findings
- To whom

## Description of Incidental Findings

Review of existing studies reveals four descriptions of incidental findings. Studies asked their participants – researchers, patients, public, geneticists, IRB chairs and members - how they would describe incidental findings. Most respondents in these studies tended to describe incidental findings under the following headings: ‘unexpected results’, ‘a finding unrelated to study aims’, ‘findings beyond study aims’ and ‘extra or unsolicited information’ (Table 1.2). The background (as geneticist, IRB chairs or medical professionals etc.) of these respondents did not play any significant role in how these respondents viewed incidental findings. Three studies, however, did not provide any information about how their participants perceived incidental findings.

## Incidental Finding Management Preference

Majority of participants in 17 studies believe that incidental findings ought to be returned to participants. For example, a significant majority of researchers (Fernandez et al., 2013, Klitzman et al., 2013), IRB chairs and members (Dressler et al., 2012, Simon et al., 2011, Williams et al., 2012), patients (Shahmirzadi et al., 2013, Master et al., 2013), public (Haga et al., 2011), geneticists^4^ (Downing et al., 2013, Green et al., 2012a, Lohn et al., 2013, Haga et al., 2012a, Driessnack et al., 2013), for example, believed in an obligation to offer incidental findings to participants. In addition, some studies (Haga et al., 2011, Dressler et al., 2012) found no statistically significant relationship between age, years of practice, country of training, race, etc., and this preference to have incidental findings returned. Two studies (Johnson et al., 2012/04, Ferriere and Van Ness, 2012), also found that very few bio-banks have policies about returning incidental findings.

## Type and Conditions for Returning Incidental Findings

The review of existing studies equally shows the various types of incidental findings, which may be returned in genomic research, as well as the conditions for returning the same. In studies conducted to assess the opinions of IRB members by Dressler and colleagues (2012), geneticists/genetic specialists by Green and colleagues (2012a) and researchers by Fernandez and colleagues (2013); most respondents in these studies believe that analytically valid (incidental) findings ought to be returned to participants. A significant majority of patients too in studies conducted by Shahmirzadi and colleagues (2013); Master and colleagues (2013); Klitzman and colleagues (2013) also want incidental findings returned if findings reveal (serious or minor) disease. Other threshold for returning incidental findings identified by a significant geneticists and other professionals in studies conducted by Downing and colleagues (2013); Lohn and colleagues (2013); Haga and colleagues (2012a) include: if findings are clinically actionable and significant; if findings are serious(treatable or untreatable) (Driessnack et al., 2013). However, some geneticists and other professionals in studies conducted by Lohn and colleagues (2013) and Haga and colleagues (2012a) add that they will be less likely to return incidental findings with unknown significance; information with a multifactorial condition; and incidental findings with personal implications.

One study (Fernandez et al., 2013) examined the duration of the obligation to return incidental findings. In the study conducted by Fernandez and colleagues (2013), a majority of researchers believe that duration of the responsibility to return incidental findings should be linked to the study period or on-going access to research result.

## Factors and Impacts of Returning of Incidental Findings

Some studies (Dressler et al., 2012, Downing et al., 2013, Driessnack et al., 2013) indicated that a significant number of their respondents believe that resource and financial implications of returning findings, psychological harm and potential legal liabilities, the difference between research and clinical care, unwillingness of researchers to promote therapeutic misconception, would influence their decision to return incidental findings. For examples, most researchers in the study conducted by Williams and colleagues (2012) generally emphasized that the purpose of research was to generate knowledge and not to provide individual results to participants. Moreover, most researches are conducted in laboratories not optimized for clinical care, thus limiting their ability to provide results for clinical purposes. However, when incidental findings, with clear health implications, are discovered, they still felt a responsibility to return such findings, since, as other respondents in Dressler and colleagues' study (2012) maintained, returning such findings will show respect for research subjects; encourage participation in future research and may help participants to take preventive measure.

## Recipient of Incidental Findings

The response to this question varied; of the 19 studies reviewed, 10 of them reported that a significant majority of their respondents (researchers, patients, public, IRB members, geneticists) favour returning incidental findings to research participants or patients. Other opinions were also expressed. One study (Master et al., 2013) which assessed the opinion of cancer patients reported that a majority of these patients wants incidental findings returned to them through their physicians. Some researchers and IRB chairs in Williams and colleagues' study (2012) indicated that family members should be informed if condition is inheritable; guardians should equally be informed if the subject is a child.

## Who Should Return Findings?

This question has not been satisfactorily studied. A few suggestions were proposed. Some of them include researchers or appropriately trained personnel. Most patients and members of public in some studies (Haga et al., 2012a, Haga et al., 2012b) want incidental findings returned by their physician or primary care giver. Most researchers in a study conducted by Klitzman and colleagues (2013) also appear to agree with this. They feel that professionals with clinical training such as genetic counsellors or medical professionals are in the best position to return such unexpected medically significant findings.

## Suggestions for Improving the Management of Incidental Findings

Very few empirical studies have reflected on this question. A majority of participants in studies (Fernandez et al., 2013, Williams et al., 2012, Klitzman et al., 2013) conducted to assess the views of researchers advice primary investigators to anticipate incidental findings before study, and offer genetic counselling before returning incidental findings. Geneticists (and researchers) in the studies conducted by Lohn and colleagues (2013), and Klitzman and colleagues (2013), suggest pre-study counselling to establish participants' preferences with regard to disclosure of incidental findings (Simon et al., 2011, Driessnack et al., 2013).

Furthermore, IRB chairs and members in the studies conducted by Simon and colleagues (2011); Dressler and colleagues (2012); Williams and colleagues (2012), want researchers to anticipate incidental findings and include a statement (or general policy) about how they would manage research results, including incidental findings in the informed consent processes. Two studies (Downing et al., 2013, Klitzman et al., 2013) revealed that some of her respondents (mainly geneticists, researchers, IRB chairs and members) want serious and preventable incidental findings returned to the participants regardless of the patient's/subject's preference not to receive the same.

## Discussion

This is the first study that will systematically review existing empirical studies on how to manage incidental findings when they arise within genomic research. This review shows that there is indeed a consensus – which cuts across board, researchers, patients, public, geneticists, IRB members, IRB chairs – that clinically actionable findings or findings with serious health conditions should be returned. Herein, one may distinguish between the different kinds of incidental results that may be generated in genomic research. Some of them will be clinically actionable (that is, the individual is at a high risk of future preventable or manageable health problem); others will be ‘not-clinically’ actionable (that is provides information for which there is no clinical action or has no implication for the individual's health status). Finally, still others will have no-known clinical significance (that is, implication of the individual's health is at the moment, unknown).

Respondents (largely researchers, geneticists, IRB chairs and members) in studies reviewed are undecided about what to do with the final category of incidental findings. What happens when clinical significance of an incidental finding becomes known, should the participant be re-contacted? Samples may have been collected years back and research participants may have changed location or moved somewhere, thus making re-contacting impracticable. And where re-identification and re-contacting are possible, because the repositories retained link to research subject's contact details, should re-contacting participants be encouraged? Re-identifying research participants for the purpose of re-contacting them to offer them findings, as mentioned in previous sections in this study, may put research participants at risks of losing their privacy, and confidentiality. Thus, what measures do we have in place to ensure that the participant's privacy is protected? Incidental findings raise questions that stretch far into the future. These questions require critical thinking which is largely missing in published empirical studies. In addition to this, the suggestion that the duration of the duty to return should be limited to study period, when the clinical implication of a result may become known in the future, may be myopic. Further empirical studies are required in these directions.

It is also instructive that the few studies which examined the opinions of researchers and research participants on ‘who should return incidental findings’ found that most respondents want such findings returned by participant's physician or primary health care giver. This directly negates the opinion expressed by Wolf and colleagues (2008b) that such findings should be returned by researchers. In addition to the fact that researchers may not be adequately trained to return such findings, burdening them with this task may distract them from achieving their study aim or objectives.

The consensus that clinically actionable finding should be returned is indeed laudable. However, one foresees a number of ethical difficulties here. In order to eliminate the possibility of false positive results and to address quality control concerns, researchers, IRB chairs and members who want such incidental findings returned also require researchers to validate or substantiate such findings in laboratories optimized for clinical care, since most genomic researches are conducted in laboratories not optimized for clinical. This recommendation has financial implications, which have not been sufficiently considered. Who will finance such validation; the researcher or the participants? Should researcher anticipate the likelihood such need to validate result in designing research protocol? Should research sponsors be burdened with financing such validation? When results are validated and confirmed to be clinically actionable; who should pay for the participant's clinical care: researcher of participants? Future empirical studies should raise these questions and bridge the existing gap.

Currently, the cost of determining the clinical significance and validity of such tests are high. This assertion has been confirmed by Solomon and colleagues (2012) in a study on ‘Incidental Medical Information in Whole-Exome Sequencing’, where they discovered that a large amount of effort (financial and personnel) was required to return only one incidental genome variant found through whole-exome sequencing. Research is an expensive enterprise. With the current advanced technology employed in genomic research, creating an obligation to return findings may make the already expensive research enterprise, more financially burdensome, or distract researchers from fulfilling research aim and objectives. Consequently, future studies need to carefully reflect on how to overcome this financial burden.

Furthermore, there is equally so much empirical uncertainty that a participant will benefit rather than harmed, from receiving incidental findings. For example how would research participants react to the news that they have serious but untreatable health condition? The knowledge may kill them faster than the condition itself. Some of these research participants may view this information as a disservice to them. Some may even think that their condition was caused by participating in research, and thus, lose faith in research enterprise. Additionally, research participants may be given information for which they are not prepared; or exposed to unnecessary treatment. All of which can subject research volunteers to significant physical and psychological harm. This is especially true for an African who prides his or her ethnic identity. Returning incidental finding of misattributed paternity to such African would cause great psychological and emotional distress to his sense or personal and ethnic identity. It is possible that no harm would result if no obligation to routinely return incidental findings is created.

Equally, full disclosure, when a participant has not indicated a preference to receive information could constitute a violation of participant's right ‘not to know’. Certain empirical studies exist that reveal, for example that some dementia patients preferred not to have been informed about their disease (Marzanski, 2000; p. 322). These dementia patients reported that disclosure breached their rights to self-determination and autonomy. Thus, to assume that ‘all’ participants want incidental findings, however clinically actionable, is a dangerous generalization. Thus, implications of returning incidental findings, for research participants and enterprise, need to be further empirically explored.

This study has limitations. The filters selected, as well as the strict criteria used for excluding and including studies for the systematic review, may have ruled out potential studies which could have been included in this review, especially studies which are neither open nor free.

Secondly, I observed that some studies had missing information on participant characteristics, which made it difficult to compare information across studies or identify whether certain characteristics such as religious views, marital status or occupation may have been responsible for the attitudes or reasons given for disclosing incidental findings. Finally, this review has pooled resources, not only from studies which assessed views or perspectives of individual human beings on ancillary findings, but empirical studies on policies and regulations on incidental findings. This has also affected how the data were organized and analysed.

Regardless of these limitations, this study makes the following recommendations:

- More empirical studies should be conducted to assess the impacts of returning incidental findings on participants and research enterprise.
- Future empirical studies should also focus on obtaining opinions of researchers, participants, IRB chairs and sponsors, research sponsors, etc., on ‘who’ should bear the financial burden of returning incidental findings.
- The general consensus to return clinically actionable findings should be further strengthened through empirical studies conducted to assess of researchers, participants, geneticists, IRB chairs and members in Africa, Europe, and Asia. At the moment, this consensus only reflects the views of Northern Americans. Given the impact of religion, culture, literacy, gender, age etc., in these regions (Africa, Europe, and Asia) can have on worldviews and attitudes to research; Africans, for example, have been described by Mbiti (1969) as notoriously religious. This point has been confirmed in more recent studies (Jegede, 2009). They (Africans) take their religion everywhere they go, to their offices, shops, kitchen, markets etc. Wherever an African man goes, there is his religion. African man and his religion are so tightly connected that without the one, the other cannot exist. As a result, it would be important to study how religion, and culture, would affect attitudes and dispositions to incidental findings in genomic research.
- Other questions, such as ‘Should hereditary findings be returned to third parties or family members?’ ‘What impacts will returning findings to third parties, have on the participants?’ also deserve greater attention in future empirical studies.

## Figures and Tables

**Table 1:** General Characteristics Table shows the general characteristics of reviewed studies.

	Author_ID	Year	Sample Size	Country
1	Ferriere and Ness	2012	10	USA
2	Green et al	2012	16	USA
3	Dressler et al	2012	31	USA
4	Simon et al	2011	34	USA
5	Haga et al	2012	45	USA

6	Haga et al	2012	21	USA
7	Downing et al	2013	50	USA
8	Williams et al	2012	53	USA
9	Fernandez et al	2013	74	Canada
10	Master et al		100	Canada

11	Driessnack et al	2013	166	USA
12	Shahmirzahi et al		200	USA
13	Lohn et al	2013	210	Canada
14	Klitzman et al	2013	254	USA
15	Lawrenz and Sobotka	2008	1023	USA

16	Haga et al	2012	1139	USA
17	Susan et al	2012	2395	USA
18	Johnson et al	2012	2395	USA
19	Goddard et al	2013		USA

**Table 2:** Description of Incidental Findings Table shows the various understanding of incidental findings. Many defined incidental finding as a finding unrelated to study aims.

Definition of Incidental Findings	Freq.(%)

Unexpected finding	3(15.79)
Unrelated to study aims	8(42.11)
Finding beyond study aim	3(15.79)
Extra information	2(10.53)
Provided no response.	3(15.79)

Total	19(100.00)

## Footnotes

1 Information management refers to the power to control the volume of information that is disclosed to or withheld from the participants or patients. G. Swaminath, “The Doctor's Dilemma: Truth Telling,” *Indian J Psychiatry* 50, no. 2 (2008).

2 This does not apply strictly to a researcher who equally has a physician/patient relationship with his research participants. In this situation, the researcher/physician has the duty to follow up on the participant's health. For the purpose of this study, our focus is on a researcher who has no physician-patient relationship with his participants.

3 The pilot test shows that certain questions, such as the duration of the duty to return, were considered by a few studies and not captured in the data extraction form. As a result, changes were made to the data extraction form reflect these important questions.

4 Clinical geneticists, genetic counsellors, laboratory geneticists, genetic professionals, etc.
